# The Role of Serum CEA and CA19-9 in Efficacy Evaluations and Progression-Free Survival Predictions for Patients Treated with Cetuximab Combined with FOLFOX4 or FOLFIRI as a First-Line Treatment for Advanced Colorectal Cancer

**DOI:** 10.1155/2019/6812045

**Published:** 2019-01-20

**Authors:** Jun Jia, Pengfei Zhang, Miaomiao Gou, Fan Yang, Niansong Qian, Guanghai Dai

**Affiliations:** ^1^Department of Oncology, Chinese People's Liberation Army General Hospital, Beijing 100853, China; ^2^Disinfection Supply Room, Chinese People's Liberation Army General Hospital, Beijing 100853, China

## Abstract

**Background:**

Previously, it was demonstrated that serum levels of tumor markers, CEA and CA19-9, correlated with chemotherapy. Consequently, it has been hypothesized that dynamic monitoring of changes in these markers may predict the shrinkage or growth of colorectal cancers. To test this hypothesis, we analyzed CEA and CA19-9 serum levels in patients with advanced colorectal cancer who received cetuximab in combination with chemotherapy. These levels were evaluated at various time points to identify their potential to serve as early efficacy predictors during treatment and early predictors of disease progression.

**Patients and Methods:**

Measurements of tumor markers, CEA and CA 19-9, in patients with metastatic colorectal cancer (*n* = 73) who received cetuximab plus folinic acid, fluorouracil, and oxaliplatin or irinotecan (FOLFOX4/FOLFIRI) as a first-line treatment at our center were retrospectively analyzed. These levels were also compared with objective responses according to the World Health Organization criteria. Initially, 65 patients had elevated CEA levels (>5 ng/ml), and 59 patients had elevated levels of CA19-9 (>37 U/ml). A total of 172 cycles and 165 cycles of computed tomography/magnetic resonance imaging observations were available for review from these two patient groups.

**Results:**

After completing three cycles of treatment, the best diagnosis of cetuximab resistance was achieved when CEA increased by 35% (efficacy, 83.33%; sensitivity, 75.41%) and when CA19-9 increased by 28% (efficacy, 80.00%; sensitivity, 84.31%). Next, the efficacy of cetuximab at the time of diagnosis (at the first imaging examination/after three cycles of treatment) was evaluated after the first cycle of chemotherapy. When CEA decreased by 60% from its baseline level, the best effective rate and sensitivity were observed (63.64% and 80.95%, respectively). Similarly, when CA19-9 was 45% lower than its baseline level, the best effective rate and sensitivity were observed (84.21% and 93.18%, respectively). To evaluate progression-free survival (PFS), levels of both CEA and CA19-9 were evaluated after the third cycle of chemotherapy. Increases of 35% and 28%, respectively, resulted in a shorter PFS period compared with the other patients (3.15 months vs. 9.10 months, respectively; *P* < 0.0001). Conversely, when the evaluation was performed after the first cycle of chemotherapy, patients exhibiting a 60% decrease in CEA and a 45% decrease in CA19-9 had a longer PFS period (11.13 months vs. 8.10 months, respectively; *P* = 0.0395).

**Conclusions:**

CEA and CA19-9 are useful indicators of therapeutic curative effect from cetuximab combined with first-line chemotherapy. These markers also helped assess cetuximab resistance and served as early predictors of initial treatment effectiveness. Furthermore, a simultaneous increase or decrease in the levels of both indicators was consistent with the observed differences in PFS.

## 1. Introduction

Colorectal cancer is the most common malignant tumor in the digestive system. In 2017, the incidence and mortality of colorectal cancer were third among all types of cancer worldwide [1]. In China, the incidence and mortality of colorectal cancer have increased in parallel with developments in the economy over the past 30 years [2]. The prognosis of colorectal cancer is closely related to cancer stage at the time of diagnosis, and approximately 30% of patients have distant metastases when they are diagnosed. However, even when surgical treatment is performed during the early stages of colorectal cancer, approximately 50% of patients will develop recurrence and metastasis. Therefore, systemic chemotherapy is an important treatment option for patients to prolong their survival and improve quality of life. Among the first-line treatment options currently available, a regimen including folinic acid, fluorouracil, and oxaliplatin (FOLFOX4) and a regimen including folinic acid, fluorouracil, and irinotecan (FOLFIRI) have been shown to improve patient progression-free survival (PFS) and overall survival (OS) [3, 4]. However, no statistically significant differences have been observed in time to progression (TTP) and OS between the two regimens [3, 4].

There have been many advances in the treatment of colorectal cancer over the past decade. Regarding molecular therapy for colorectal cancer, cetuximab has become widely used as a competitive inhibitor of epidermal growth factor receptor (EGFR). Van Cutsem et al. and Borner et al. both reported that cetuximab combined with FOLFIRI as a first-line treatment for *RAS* wild-type metastatic colorectal cancer significantly increased the response rate and prolonged PFS [5, 6]. Furthermore, cetuximab in combination with chemotherapy as a first-line treatment for advanced colorectal cancer has shown good safety and efficiency in clinical applications in recent years, with significant improvements in patients' PFS and OS observed [7–10].

To evaluate curative effects, imaging examinations (usually computed tomography (CT) or magnetic resonance imaging (MRI)) and Response Evaluation Criteria in Solid Tumors (RECIST) are generally used as the basis and standard for clinical treatments worldwide [11]. However, these imaging methods are relatively expensive, and patients have expressed concern regarding the potential harm from radiation during imaging. As a result, poor patient compliance with imaging examinations has been observed. In addition, for some metastatic solid tumors, such as abdominal lymph node metastases, it is difficult to apply these imaging methods. Additional assessments can also be needed for certain clinical applications. In contrast, monitoring of tumor markers in serum is relatively simple and inexpensive, it is associated with good sensitivity and specificity, and it is highly reproducible.

For patients affected by different types of tumors, tumor-associated markers are usually elevated. During treatment, these tumor markers often undergo dynamic changes. Many studies have shown that changes in the levels of two tumor markers, in particular CEA and CA19-9, are closely related to curative effects and the prognosis of advanced colorectal cancer [12–20]. However, roles for CEA and CA19-9 in evaluating efficacy and in predicting disease progression during cetuximab treatment combined with chemotherapy have not been reported. Based on the correlation previously described between CEA and CA19-9 and chemotherapy, it is predicted that monitoring of changes in the levels of these markers may predict tumor shrinkage or growth. To test this hypothesis, patients with advanced colorectal cancer who received cetuximab in combination with chemotherapy within the previous five years at our center were retrospectively examined in regard to their levels of CEA and CA19-9. Changes in these levels were then evaluated for their potential to serve as early efficacy predictors during the course of treatment and as early predictors of disease progression.

## 2. Data and Methods

### 2.1. Clinical Data

This study reviewed patients with advanced colorectal cancer who were treated with a first-line treatment regimen of CET+FOLFOX4/FOLFIRI (see Section 2.2 for treatment details) between June 2012 and June 2017 at our center (Table 1). There were 73 patients totally. The number of primary lesions located in the colon and in the rectum was 60 and 13, respectively. The number of patients having increased initial CEA (>5 ng/ml) was 65, and the number of patients having increased initial CA19-9 (>37 U/ml) was 59. In the cohort, 59 patients had one metastasis, and the others had multiple metastases. In the treatment plan, there were 41 patients treated with cetuximab+FOLFOX and 32 treated with cetuximab+FOLFIRI. The inclusion criteria were as follows: a histopathological diagnosis of colorectal cancer, no mutations in either *KRAS* or *NRAS*, patients with advanced colorectal cancer (TNM staging IV) who could not undergo local surgery or radiotherapy, an Eastern Cooperative Oncology Group (ECOG) score of 0–2, an expected life period of >3 months, and normal results for electrocardiography, routine blood panel, and liver and kidney function. The exclusion criteria were brain and bone metastases and other malignant diseases which could impair patient survival.

### 2.2. Therapeutic Method

According to the guidelines established by the National Comprehensive Cancer Network, the dose scheme for the FOLFOX4 regimen includes the following: oxaliplatin at a dose of 85 mg/m^2^ via intravenous (i.v.) infusion (d1), leucovorin at a dose of 400 mg/m^2^ via i.v. infusion (d1), fluorouracil at a dose of 400 mg/m^2^ via i.v. infusion (d1), and fluorouracil at a dose of 3000 mg/m^2^ via continuous pump for 46 h. The dose scheme for the FOLFIRI regimen includes the following: irinotecan at a dose of 180 mg/m^2^ via i.v. infusion (d1), leucovorin at a dose of 200 mg/m^2^ via i.v. infusion (d1), fluorouracil at a dose of 400 mg/m^2^ via i.v. infusion (d1), and fluorouracil at a dose of 3000 mg/m^2^ via continuous pump for 46 h. The dose of cetuximab administered was 500 mg/m^2^ for 14 days per cycle (i.v.).

### 2.3. Efficacy Evaluation

All patients underwent a CT/MRI examination. Blood tests were performed to detect serum levels of CEA and CA19-9 within two weeks of the start of treatment (representing baseline values). Both tumor markers were subsequently retested within 2 d of starting a new cycle of treatment. Abdominal enhanced CT/MRI scans and noncontrast lung CT scans were reviewed each time three cycles of chemotherapy were completed.

#### 2.3.1. Clinical Efficacy Evaluation

According to the RECIST criteria, complete remission (r-CR) was defined as the disappearance of all target lesions, partial remission (r-PR) was defined as the sum of the maximum diameter of the baseline lesions that decreased by at least 30%, and disease progression (r-PD) was defined as the sum of the maximum diameter of the baseline lesions that increased by at least 20% or the appearance of new lesions. Furthermore, when the sum of the maximum diameter of the baseline lesions was reduced (yet not to the point of PR) or, conversely, if an increase was observed that was not to the point of PD, the patient was considered to have reached a stable disease state (r-SD). Disease control (r-DCR) was calculated as r − CR + r − PR + r − SD.

Serum levels of CEA and CA19-9 were determined by using an enzyme immunoassay test kit with 5 ng/ml and 37 U/mL considered the upper limits of normal for each enzyme, respectively, according to the manufacturer's instructions. Serum levels of CEA and CA19-9 were measured every cycle before chemotherapy. In our Oncology Unit, tumor marker monitoring is routinely performed in order to identify patients who could potentially benefit from the treatment. In this context, CEA and CA19-9 data were evaluated if they were collected within one month of the start of treatment and also if they were collected approximately 2–4 weeks before PD was established based on imaging scans. Biomarker monitoring was performed every two weeks until disease progression was first documented.

For all cycles involving a reduction in tumor marker levels, sensitivity was defined as true positives/(true positives + false negatives), specificity was defined as true negatives/(true negatives + false positives), positive predictive values were defined as true positives/(true positives + false positives), and negative predictive values were defined as true negatives/(true negatives + false negatives).

### 2.4. Statistical Methods

SPSS 26.0 software was used to perform statistical analyses. Differences between groups were compared according to the chi-square test, cut-off values were determined with receiver operator characteristic (ROC) curves, and univariate survival analyses were performed with Kaplan-Meier survival curves. *P* values less than 0.05 were considered to indicate statistically significant differences.

## 3. Results

### 3.1. Comparison of RECIST Standards with CEA and CA19-9 Standards

According to the admission criteria for this study, 73 patients were retrospectively analyzed. Among these patients, 65 patients and 59 patients had elevated baseline levels of CEA or CA19-9, respectively. For these two groups, their elevated levels for each treatment cycle were compared with those for the previous cycle. An increase in levels was considered to indicate tumor progression, while the absence of an increase was considered to indicate disease control. Changes in CEA and CA19-9 levels for each cycle were also compared with the RECIST criteria, with 172 cycles and 165 cycles of CT/MRI observations available for each group, respectively. Based on these data, the sensitivity of CEA in assessing disease control rate was 94.2%, its specificity was 71.4%, its positive predictive value was 90.0%, and its negative predictive value was 81.9% (Table 2). For CA19-9, the corresponding values were 91.5%, 83.9%, 94.4%, and 77.0%, respectively (Table 2). When levels of both CEA and CA19-9 were considered, the sensitivity increased to 97.4%, the specificity was 93.0%, the positive predictive value was 96.8%, and the negative predictive value was 91.7% (Table 2). Taken together, these results indicate that it is feasible to evaluate treatment efficacy by monitoring changes in serum levels of CEA and CA19-9. Furthermore, treatment resistance can potentially be predicted based on changes in the levels of these indicators at specific time points.

### 3.2. CEA and CA19-9 Predict Cetuximab Resistance after Every Three Cycles of Treatment

**Figure 1:**
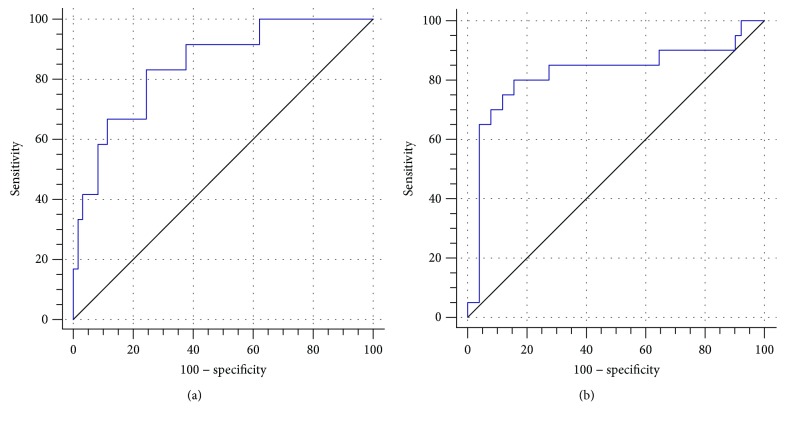
ROC curves for CEA (a) and CA19-9 (b) were compared with RECIST standards to determine the cut-off values for each for cetuximab resistance.

When changes in the serum levels of CEA and CA19-9 after every three cycles of treatment were compared with the RECIST criteria (Figure 1), the efficacy and sensitivity of detecting cetuximab resistance were the highest when the level of CEA increased by 35%. For this increase, the corresponding efficacy and sensitivity rates were 83.33% and 75.41%, respectively. The area under the ROC curve (AUC) was 0.85 (95% confidence interval (CI): 0.74–0.92). Similarly, when the serum level of CA19-9 increased by 28%, the efficacy and sensitivity of detecting cetuximab resistance were 80.00% and 84.31%, respectively, and the AUC was 0.82 (95% CI: 0.71–0.90).

### 3.3. CEA and CA19-9 Predict Cetuximab Effectiveness after the First Cycle of Treatment

**Figure 2:**
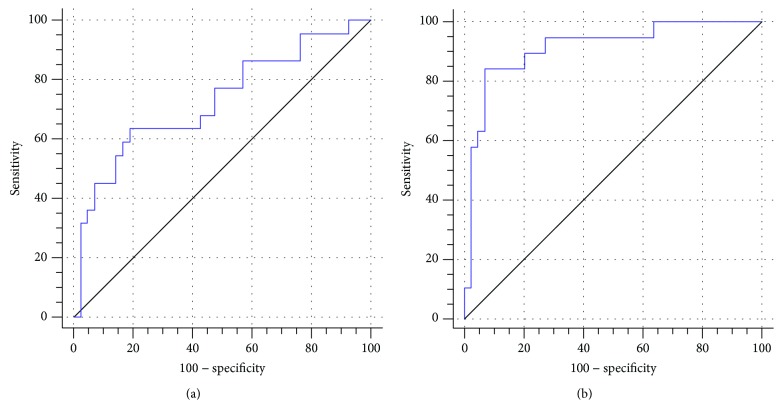
ROC curves for CEA (a) and CA19-9 (b) were compared with RECIST standards to determine the cut-off values for effective cetuximab treatment.

When serum levels of CEA and CA19-9 were examined after the first cycle of chemotherapy (Figure 2), a decrease in the level of CEA by 60% from baseline provided an effective and sensitive prediction of cetuximab's efficacy after three cycles of treatment (i.e., at the first imaging examination). The effective rate and sensitivity were 63.64% and 80.95%, respectively, with an AUC value of 0.73 (95% CI: 0.60–0.83). Similarly, when a decrease of 45% from baseline was observed for the level of CA19-9, the effective rate and sensitivity for diagnosing cetuximab's efficacy were 84.21% and 93.18%, respectively, with an AUC value of 0.91 (95% CI: 0.82–0.97).

### 3.4. CEA and CA19-9 Prediction of PFS

**Figure 3:**
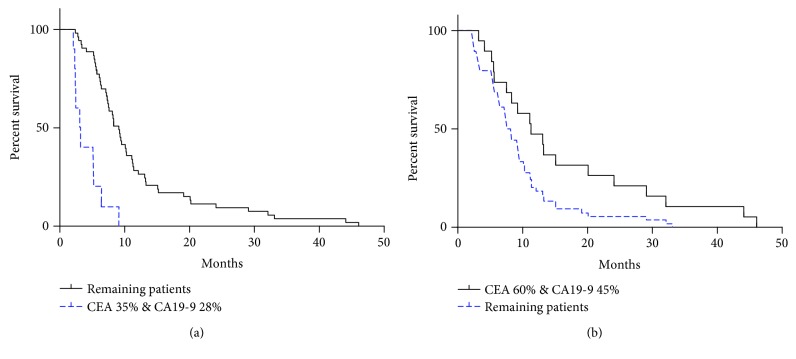
Comparisons of PFS. (a) After the third cycle of treatment, patients with a 35% increase in CEA and a 28% increase in CA19-9 had a shorter PFS compared with the remaining patients. (b) After the first cycle of treatment, patients with a decrease in CEA by 60% and a decrease in CA19-9 by 45% had a longer PFS compared with the remaining patients.

When serum levels of CEA and CA19-9 were examined after the third cycle of chemotherapy, patients' PFS was also examined (Figure 3). When the level of CEA increased by 35% and the level of CA19-9 increased by 28%, the PFS period was shorter than that of the other patients (3.15 months versus 9.10 months, respectively; *P* < 0.0001). In comparison, when the levels of these markers were examined after the first cycle of chemotherapy, the patients exhibiting a 60% decrease in their level of CEA and a 45% decrease in their level of CA19-9 exhibited a longer PFS period than the other patients (11.13 months and 8.10 months, respectively; *P* = 0.0395).

### 3.5. Characteristics of the Patients with Shorter PFS

When we further examined the characteristics of the patients with a shorter PFS period (Table 3) (e.g., those with a 35% increase in CEA level and a 28% increase in CA19-9 level after the third cycle of chemotherapy), most of these patients had rectal cancer with multiple metastases. Furthermore, when metastases were only present in the peritoneum and local abdomen, the PFS of these patients was worse than that of patients with other sites of metastasis. There was no significant difference in PFS according to gender, although the small number of cases prevented a statistical analysis from reaching significance. However, these results provide insight into which patients may be better suited for receiving cetuximab treatment, and this is the focus of our subsequent studies which include a larger number of cases.

## 4. Discussion

Imaging examinations are often used to assess the therapeutic effects of drug treatments for solid tumors. However, in our experience, a large proportion of patients have poor adherence to imaging examinations due to their high cost and/or fear of radiation injury. A proportion of patients also have metastases which are difficult to measure, and this can make assessments difficult. Previously, we reported that tumor markers can provide significant guidance regarding tumor progression and resistance to traditional anticancer drugs. Moreover, the detection of tumor markers can be relatively simple and inexpensive and have minimal adverse effects. It remains to be confirmed whether tumor markers can also be used to evaluate the efficacy of cetuximab in combination with chemotherapy.

With advances being made in the development of various cancer treatment methods, particularly with the rapid development of anticancer drugs, the corresponding requirements for evaluating therapeutic effects have become increasingly diversified and precise. However, various studies [21–23] have described that for targeted therapy and immunotherapy treatments, there is usually a delay before their therapeutic effects are evaluated. Similarly, if tumor volume is evaluated with conventional CT or MRI, tumor size may not change until after a few cycles of continuous treatment have been completed or the tumor only appears as a hollow entity. As a result, evaluation standards to assess the curative effect on traditional solid tumors appear to be increasingly incompatible with providing a comprehensive assessment and early prediction of the therapeutic effect. Moreover, they often need to be supplemented and supported by other methods. RECIST standards state that tumor markers can be used as auxiliary indicators for evaluating curative effects [24]. For example, after retrospectively analyzing 531 cases of ovarian cancer, Duffy [25] highlights that continuous monitoring of CA125 levels was able to reflect the curative effect of ovarian cancer chemotherapy in 89% of the cases examined. In addition, a decline in CA125 levels was found to be related to the curative effect and the prognosis of ovarian cancer. A number of studies have also shown that levels of CEA and CA19-9 are closely related to the curative effect of chemotherapy and large doses of fluorouracil and calcium folinate as treatments for advanced and metastatic colorectal cancer, respectively [16–18]. Previously, the tumor markers used have mostly included individual indicators, and these were characterized by low specificity and poor sensitivity. Subsequently, the use of multiple indicators was proposed to monitor and evaluate the curative effects of cancer treatments [19]. In our retrospective analysis, CEA and CA19-9 exhibited good sensitivity and specificity in evaluating the curative effect of cetuximab in combination with first-line chemotherapy. Moreover, the use of both markers was better than the use of either marker individually. When the proportion of patients with elevated levels at baseline subsequently exhibited a further increase in marker levels, the sensitivity of CEA in assessing disease control rate was 94.2% (specificity, 71.4%), and for CA19-9, it was 91.5% (specificity, 83.9%). When both markers increased, which was only observed in a small number of patients, the sensitivity and specificity of evaluating disease control were higher, with rates of 97.4% and 93.0%, respectively. These results demonstrate that CEA and CA19-9 can be combined as indicators of curative effect during chemotherapy, although this remains to be confirmed in a larger sample number. Many studies have shown that CEA and CA19-9 have predictive and diagnostic effects on survival period analysis and disease control for patients who receive bevacizumab or cetuximab in combination with oxaliplatin- and irinotecan-based chemotherapy [20, 26–28]. In addition, other studies have shown that when levels of CEA decrease by more than 50% from baseline, a significant disease in remission rates is observed [23]. To date, levels of CEA and CA19-9 have rarely been studied in relation to treatment resistance and PFS for cetuximab treatments, primarily due to the high cost of cetuximab treatments. However, most patients are very interested in the efficacy of the treatment drug they are prescribed. Thus, the ability to provide an early assessment of drug efficacy during a patient's treatment process would be very helpful. Consequently, the retrospective analysis we performed included a comparison of tumor marker levels for each cycle of treatment and in relation to the imaging results obtained. When CEA values increased by 35% or CA19-9 levels increased by 28%, compared with those in the previous cycle, the probability of cetuximab resistance was considered to be high. In addition, the results from imaging examinations were used to determine whether disease progression had occurred. In one patient, both CEA and CA19-9 levels increased in parallel compared to those in the previous cycle. For this patient, the PFS period was shorter (3.15 months). When levels of CEA and CA19-9 were reduced by 60% or 45% from baseline, respectively, after the first dose of cetuximab, these changes were predictive of the effectiveness of cetuximab observed at the first imaging examination (e.g., after three cycles of treatment). Furthermore, if both levels decreased to less than the baseline level, the patients had a longer PFS period (11.13 months).

In summary, we examined different time points during patients' treatments for advanced colorectal cancer and evaluated the efficacy of monitoring changes in serum levels of CEA and CA19-9. The present results demonstrate that both markers can be used as indicators for evaluating the curative effect of cetuximab administered in combination with first-line chemotherapy. Specifically, monitoring of these markers after every three cycles of treatment facilitated assessments of cetuximab resistance. Moreover, these levels served as early predictors of the effectiveness of the initial treatment. The present results also demonstrate the potential for a simultaneous increase or decrease in CEA and CA19-9 levels to predict PFS after the first three cycles of treatment. However, due to the small sample size of this study, the relatively strict screening conditions that were used, and the analysis of data that were only obtained from a single center, it is possible that the patients selected for this study were biased toward having higher baseline levels of the indicators examined. Therefore, further studies are needed, especially prospective analysis studies. Additionally, it was observed that the patients who did not have higher tumor marker levels at baseline exhibited changes in marker levels that were not associated with disease progression. Consequently, it is possible that the cut-off values that were used to assess changes from baseline levels are not adequate for all patients throughout their course of disease, and this needs to be further investigated.

## Figures and Tables

**Table 1:** Baseline patient characteristics for our cohort (*n* = 73).

Characteristics	No. of patients
Age (years)	
Median	63
Range	47–72
Gender	
Male	42
Female	31
ECOG score	
0	40
1	29
2	4
Primary tumor	
Colon	60
Rectum	13
Metastasis site	
Liver	41
Lung	28
Local abdominal mass	10
Peritoneum	10
No. of metastatic sites	
1	59
>1	14
Chemotherapy regimen	
Cetuximab+FOLFOX	41
Cetuximab+FOLFIRI	32
Elevated markers at baseline	
CEA (>5 ng/ml)	65
CA19-9 (>37 U/ml)	59

**Table 2:** Efficacy of using CEA and CA19-9 as tumor markers.

Evaluation parameter	CEA	CA19-9
Sensitivity	162/172 (94.2%)	151/165 (91.5%)
Specificity	45/63 (71.4%)	47/56 (83.9%)
Positive predictive value	162/180 (90.0%)	151/160 (94.4%)
Negative predictive value	45/55 (81.9%)	47/61 (77.0%)

If the levels of CEA or CA19-9 were higher than those in the previous cycle or higher than the normal reference value, tumor progression was indicated. The opposite pattern indicated disease control. A total of 162 cycles and 151 cycles of CT/MRI observations were compared with the RECIST standards. There were 45 and 47 false-negative cycles, 10 and 14 true-negative cycles, and 18 and 9 false-positive cycles, respectively, in each case.

**(a):** 

Characteristics	CEA		*P* value
	>35%	≤35%	
Gender			1.000
Male	10	32	
Female	8	23	
Primary tumor			0.022
Colon	7	38	
Rectum	11	15	
No. of metastatic sites			
1	10	49	
>1	8	6	
Only liver	2	30	
Only lung	3	14	
Only local abdominal mass	2	3	
Only peritoneum	3	2	

**(b):** 

Characteristics	CA19-9		*P* value
	>28%	≤28%	
Gender			0.774
Male	8	34	
Female	7	24	
Primary tumor			0.075
Colon	6	39	
Rectum	9	19	
No. of metastatic sites			
1	8	51	
>1	7	7	
Only liver	2	30	
Only lung	2	15	
Only local abdominal mass	2	3	
Only peritoneum	2	3	

**(c):** 

Characteristics	CEA > 35% & CA19-9 > 28%		*P* value
	Yes	No	
Gender			1.000
Male	5	37	
Female	4	27	
Primary tumor			0.078
Colon	3	42	
Rectum	6	22	
No. of metastatic sites			
1	5	54	
>1	4	10	
Only liver	1	31	
Only lung	1	16	
Only local abdominal mass	1	4	
Only peritoneum	2	3	

## Data Availability

(1) The data is a clinical statistic data. (2) The (clinical statistic) data used to support the findings of this study are available from the corresponding author upon request. (3) The above data are the foreshadowing of our next research. So requests for access to these data should be made to Jun Jia (jiaj1234@126.com) or Niansong Qian (kyotomed@foxmail.com).

## References

[1] Siegel R. L., Miller K. D., Jemal A. (2016). Cancer statistics, 2016. *CA: A Cancer Journal for Clinicians*.

[2] Chen W., Zheng R., Baade P. D. (2016). Cancer statistics in China, 2015. *CA: A Cancer Journal for Clinicians*.

[3] Tournigand C., Achille E., Lledo G. (2000). FOLFIRI followed by FOLFOX or FOLFOX followed by FOLFIRI in metastatic colorectal cancer (MCRC). Preliminary results of a randomized phase III study of the GERCOR. *Annals of Oncology*.

[4] Tournigand C., André T., Achille E. (2004). FOLFIRI followed by FOLFOX6 or the reverse sequence in advanced colorectal cancer: a randomized GERCOR study. *Journal of Clinical Oncology*.

[5] Van Cutsem E., Nowacki M., Lang I. (2007). Randomized phase III study of irinotecan and 5-FU/FA with or without cetuximab in the first-line treatment of patients with metastatic colorectal cancer (mCRC): the CRYSTAL trial. *Journal of Orthopaedic Research*.

[6] Borner M., Koeberle D., von Moos R. (2008). Adding cetuximab to capecitabine plus oxaliplatin (XELOX) in first-line treatment of metastatic colorectal cancer: a randomized phase II trial of the Swiss Group for Clinical Cancer Research SAKK. *Annals of Oncology*.

[7] Ciardiello F., Lenz H. J., Kohne C. H. (2014). Treatment outcome according to tumor *RAS* mutation status in CRYSTAL study patients with metastatic colorectal cancer (mCRC) randomized to FOLFIRI with/without cetuximab. *Journal of Clinical Oncology*.

[8] Bokemeyer C., Köhne C. H., Ciardiello F. (2015). FOLFOX4 plus cetuximab treatment and *RAS* mutations in colorectal cancer. *European Journal of Cancer*.

[9] van Cutsem E., Köhne C. H., Folprecht G., Guenther S., Beier F., Papamichael D. (2016). Efficacy and safety of first-line cetuximab + FOLFIRI in older and younger patients (pts) with *RAS* wild-type (wt) metastatic colorectal cancer (mCRC) in the CRYSTAL study. *Journal of Clinical Oncology*.

[10] Köhne C. H., Poston G., Folprecht G. (2016). FOLFIRI plus cetuximab in patients with liver-limited or non-liver-limited RAS wild-type metastatic colorectal cancer: a retrospective subgroup analysis of the CRYSTAL study. *European Journal of Surgical Oncology*.

[11] Watanabe H., Okada M., Kaji Y. (2009). New response evaluation criteria in solid tumours-revised RECIST guideline (version 1.1). *Gan to Kagaku Ryoho Cancer & Chemotherapy*.

[12] Gronlund B., Høgdall C., Hilden J., Engelholm S. A., Høgdall E. V. S., Hansen H. H. (2004). Should CA-125 response criteria be preferred to response evaluation criteria in solid tumors (RECIST) for prognostication during second-line chemotherapy of ovarian carcinoma?. *Journal of Clinical Oncology*.

[13] Bast R. C., Xu F. J., Yu Y. H., Barnhill S., Zhang Z., Mills G. B. (1998). CA 125: the past and the future. *International Journal of Biological Markers*.

[14] Tas F., Karabulut S., Ciftci R. (2014). Serum levels of LDH, CEA, and CA19-9 have prognostic roles on survival in patients with metastatic pancreatic cancer receiving gemcitabine-based chemotherapy. *Cancer Chemotherapy and Pharmacology*.

[15] Bac D. J., Kok T. C., van der Gaast A., Splinter T. A. W. (1991). Evaluation of CA19-9 serum levels for monitoring disease activity during chemotherapy of pancreatic adenocarcinoma. *Journal of Cancer Research and Clinical Oncology*.

[16] Mayer R. J., Garnick M. B., Steele G. D., Zamcheck N. (1978). Carcinoembryonic antigen (CEA) as a monitor of chemotherapy in disseminated colorectal cancer. *Cancer*.

[17] Ferrone C. R., Finkelstein D. M., Thayer S. P., Muzikansky A., Castillo C. F. D., Warshaw A. L. (2006). Perioperative CA19-9 levels can predict stage and survival in patients with resectable pancreatic adenocarcinoma. *Journal of Clinical Oncology*.

[18] Hanke B., Riedel C., Lampert S. (2001). CEA and CA 19-9 measurement as a monitoring parameter in metastatic colorectal cancer (CRC) under palliative first-line chemotherapy with weekly 24-hour infusion of high-dose 5-fluorouracil (5-FU) and folinic acid (FA). *Annals of Oncology*.

[19] Dacic S. (2007). Molecular profiling of lung carcinoma: identifying clinically useful tumor markers for diagnosis and prognosis. *Expert Review of Molecular Diagnostics*.

[20] Petrioli R., Licchetta A., Roviello G. (2012). CEA and CA19.9 as early predictors of progression in advanced/metastatic colorectal cancer patients receiving oxaliplatin-based chemotherapy and bevacizumab. *Cancer Investigation*.

[21] King D. M. (2005). The radiology of gastrointestinal stromal tumours (GIST). *Cancer Imaging*.

[22] di Giacomo A. M., Danielli R., Guidoboni M. (2009). Therapeutic efficacy of ipilimumab, an anti-CTLA-4 monoclonal antibody, in patients with metastatic melanoma unresponsive to prior systemic treatments: clinical and immunological evidence from three patient cases. *Cancer Immunology Immunotherapy*.

[23] Yang A., Xiang F., Jia S. (2014). Clinical considerations for therapeutic cancer vaccines: an introduction to FDA guidance for industry. *Chinese Journal of Cancer Biotherapy*.

[24] Aaronson N. K., Meyerowitz B. E., Bard M. (1991). Quality of life research in oncology. Past achievements and future priorities. *Cancer*.

[25] Duffy M. J. (2001). Clinical uses of tumor markers: a critical review. *Critical Reviews in Clinical Laboratory Sciences*.

[26] Tsai H. L., Chang Y. T., Chu K. S. (2008). Carcinoembryonic antigen in monitoring of response to cetuximab plus FOLFIRI or FOLFOX-4 in patients with metastatic colorectal cancer. *The International Journal of Biological Markers*.

[27] Prager G. W., Braemswig K. H., Martel A. (2014). Baseline carcinoembryonic antigen (CEA) serum levels predict bevacizumab-based treatment response in metastatic colorectal cancer. *Cancer Science*.

[28] Michl M., Stintzing S., Fischer von Weikersthal L. (2016). CEA response is associated with tumor response and survival in patients with KRAS exon 2 wild-type and extended RAS wild-type metastatic colorectal cancer receiving first-line FOLFIRI plus cetuximab or bevacizumab (FIRE-3 trial). *Annals of Oncology*.

